# Groningen Frailty Indicator–Chinese (GFI-C) for pre-frailty and frailty assessment among older people living in communities: psychometric properties and diagnostic accuracy

**DOI:** 10.1186/s12877-022-03437-1

**Published:** 2022-10-07

**Authors:** Emma Yun Zhi Huang, Jasmine Cheung, Justina Yat Wa Liu, Rick Yiu Cho Kwan, Simon Ching Lam

**Affiliations:** 1 Division of Social Worker, Zhongshan Polytechnic, No.25 Bo’ai 7th Road, East District, Zhongshan City, Guangdong Province People’s Republic of China; 2 School of Nursing, Tung Wah College, Ma Kam Chan Memorial Building, 31 Wylie Road, Hong Kong SAR, China; 3 School of Nursing, The Hong Kong Polytechnic University, 11 Yuk Choi Road, Hung Hom, Hong Kong SAR, China; 4 Integrative Health Centre, Tung Wah College, Cheung Chin Lan Hong Building, 98 Shantung Street, Hong Kong SAR, China

**Keywords:** Pre-frailty, Frailty, Adaptation, Validation, Factor analysis, Psychometric property, Diagnostic accuracy

## Abstract

**Background:**

The early identification of pre-frailty and frailty among older people is a global priority because of the increasing incidence of frailty and associated adverse health outcomes. This study aimed to validate the Groningen Frailty Indicator-Chinese (GFI-C), a widely used screening instrument, and determine the optimal cut-off value in Chinese communities to facilitate pre-frailty and frailty screening.

**Methods:**

This methodological study employed a cross-sectional and correlational design to examine the psychometric properties of GFI-C, namely, internal consistency, stability, and concurrent and construct validities. The appropriate cut-off values for pre-frailty and frailty screening in the receiver-operating characteristic (ROC) curve were determined through sensitivity and specificity analysis.

**Results:**

A total of 350 community older people had been assessed and interviewed by a nurse. The GFI-C showed satisfactory internal consistency (Cronbach’s α = 0.87) and two-week test-retest reliability (intra-class correlation coefficient = 0.87). Concurrent validity (r = 0.76, *p* < 0.001) showed a moderate correlation with Fried’s frailty phenotype. The known-groups method, hypothesis testing and confirmatory factory analysis (three-factor model; χ^2^/df = 2.87, TLI = 0.92, CFI = 0.93, GFI = 0.92, RMR = 0.014; RMSEA = 0.073) were suitable for the establishment of construct validity. Based on the ROC and Youden’s index, the optimal cut-off GFI-C values were 2 (sensitivity, 71.5%; specificity, 84.7%) for pre-frailty and 3 for frailty (sensitivity, 88.2%; specificity, 79.6%).

**Conclusions:**

The result indicated that GFI-C is a reliable and valid instrument for pre-frailty and frailty screening among older Chinese people in communities. For optimal diagnostic accuracy, the cut-off values of 3 for frailty and 2 for pre-frailty are recommended.

**Supplementary Information:**

The online version contains supplementary material available at 10.1186/s12877-022-03437-1.

## Introduction

The worldwide prevalence of frailty and pre-frailty among community-dwelling older people has been reported in the systematic review and meta-analysis of Ofori-Asenso [1]. China has the world’s largest rapidly ageing population [2–5]. The weighted incidence rate of frailty reaches 60.6 per 100 person-years, and regional differences in incident frailty have been observed (44.8 per 1000 person-years in the southeast; 93.0 per 1000 person-years in the northwest) [2, 6, 7]. The overall pooled prevalence of frailty and pre-frailty total 10% and 43%, respectively, among community-dwelling older people living in urban areas [7, 8]. Given the high prevalence of frailty and its associated health consequences, increasing demands for healthcare services have imposed a considerable burden on healthcare cost and resource utilisation [6, 9, 10].

Given the high prevalence of frailty and related burden of adverse health outcomes for frail older people, the early identification of frailty, especially for community-dwelling older people, should be the priority in the primary care network [11–15]. A longitudinal study conducted in China [13] showed that 70% of frail individuals exhibited no change in their frailty status, and 7.8% manifested an improved status after changes in lifestyle factors. Modifiable risk factors and the reversible state of frailty amplify the need for early screening of frailty status.

Using validated tools to identify pre-frail/frail older people is an essential step in estimating the community need and hence the formulation of preventative services [16–18]. Various frailty screening tools are available, but no tool has been developed for the Asia-Pacific region [12, 14]. Multiple frailty screening tools are non-interchangeable and transferrable across different countries [10, 19, 20]. Discrepancies in cultural adaptation, particularly potential incongruities in translating languages and concepts of frailty, the inadequate psychometric analysis of reliability and construct validity of adapted tools, and the lack of cut-off points adjusted for Chinese populations may contribute to variability in prevalence estimates [21, 22]. Another issue compromising prevalence estimates is whether unidimensional physical frailty phenotype [23] and multidimensional phenotype should be used in frailty screening. The evolving definition of frailty encompasses physical, social, cognitive, psychological, and nutritional domains [24, 25]. The effectiveness and feasibility of existing frailty screening tools, such as administration time and questionnaire administration method for frailty assessment, are rarely evaluated [14, 26]. Considering the rapid ageing population problem in China, a brief and valid frailty measurement for frailty prevalence screening on older people is needed because of the large population [7, 14]. However, the Frailty Index or Fried’s Phenotype is the world’s most commonly used frailty measurement; it was designed for clinical use with the involvement of healthcare professionals and is time-consuming [19, 27]. Hence, it was costly and impracticable for cross-sectional large-population prevalence research [14, 28–31].

For estimating the frailty of older people in the community setting, the self-reported measurement was recommended and deemed appropriate [14]. The Groningen Frailty Indicator (GFI) is a 15-item self-reported screening tool that includes four domains of frailty: physical components (mobility, comorbidity, physical energy, vision, and hearing), psychological component (depressed mood and anxiety feelings), cognitive component (cognition) and social component (loneliness) [24, 32–34]. A score of 1 or 0 is assigned to any ‘yes/sometimes’ or ‘no’ response, respectively. A total score of 4 or higher that represented moderate to severe frailty was validated in the Netherlands [24, 35]. The self-reported style of the GFI shows good feasibility, whereby 84% of older people in the Netherlands (who can read English) had no difficulty completing the GFI [24]. Factor analysis indicated the three-factor model as an appropriate internal structure (i.e. daily activities, psychosocial functioning, and health problems) and explained 50.6% of the variance [24, 36]. Satisfactory internal consistency, scalability, and criterion validity were reported for the ‘Daily Activities’ (Cronbach’s α = 0.81, Hs = .84; *r* = −.62) and ‘Psychosocial Functioning’ subscales (Cronbach’s α = 0.80, Hs = .35; *r* = −.48). Marginal internal consistency, acceptable scalability, and criterion validity were reported for the ‘Health Problems’ subscale (Cronbach’s α = .57, Hs = .35; *r* = −.48) [36]. The GFI has been translated from English into Chinese (i.e., named GFI-C) using Brislin’s model, and the semantic equivalences between the source language and the back-translated version were established [20]. Guided by 12 bilingual healthcare experts, the item (83–100%) and scale (86–100%) level semantic equivalences were satisfactory, and the content validity index was 98%. The older participants (50% illiterate) can accept and comprehend most items (100% acceptance, 97% comprehensibility) [20]. However, a comprehensive validation of GFI-C for measuring frailty and pre-frailty of the Chinese older population has not yet been established. Hence, this methodological study was conducted to report the psychometric properties of GFI-C and determine the optimal cut-off values for screening the frailty and pre-frailty older people in the community.

## Methods

### Design

This methodological study employed a cross-sectional and correlational design. Table 1 shows the testing properties, statistical methods, and sample sizes of psychometric and diagnostic accuracy tests.

**Table 1:** Testing and statistical methods of psychometric testing and diagnostic accuracy test

Psychometric Properties	Methods of Testing	Statistical Method and Cut-Off Standard	Testing Samples
Reliability			
Internal consistency	Cronbach’s method	Cronbach’s α statistic, > 0.7 = satisfactory	All 350 older people
Stability	Two-week test-retest reliability	Intra-class correlation coefficient (ICC), > 0.75 = satisfactory	A subgroup of at least 50 older people (Giraudeau & Mary, 2001)
Validity			
Criterion-related validity	Concurrent validity: correlating GFI-C with the Fried’s frailty phenotype	Pearson moment–product correlation coefficient, r ≥ 0.7 & < 0.9 = satisfactory	All 350 older people
Construct validity			
1. Known-groups method	Comparing the GFI-C of older people in the community and long term care facility	1. Independent sample t-test, significant result = satisfactory	All 350 older people
2. Hypothesis testing:	Correlating the frailty (GFI-C) with cognitive level (AMT) and physical ability (SBI)	2. Pearson moment-product correlation coefficient, r > 0.5 = satisfactory	All 350 older people
3. Factor analysis		3. Confirmatory factor analysis χ^2^/df < 5.0, TLI > 0.90, CFI > 0.90, GFI > 0.90, RMR < 0.05, RMSEA ≤ 0.08.	All 350 older people
Diagnostic accuracy test			
Sensitivity and specificity analysis	Comparing GFI-C results with the Fried’s frailty phenotype results	The receiver-operating characteristic (ROC) curve, sensitivity and specificity > 0.70	All 350 older people
Discriminative properties of the diagnostic accuracy		The area under the curve (AUC), AUC > 0.70	

*GFI-C* Groningen Frailty Indicator – Chinese, *AMT* Abbreviated Mental Test, *SBI* Simplified Barthel Index, *TLI* Tucker–Lewis Index, *CFI* Comparative Fit Index, *GFI* Goodness-of-fit Index, *RMR* Root Mean Square, *RMSEA* Root Mean Square Error of Approximation

### Study participants

The sample size was estimated based on the sensitivity or specificity in phase 2, and Buderer’s formula [37] was used. The prevalence of frailty was set at 9.9%, in accordance with the latest literature regarding community-dwelling older people [2]. A conservative sample size of 350 was adopted. From November 2017 to March 2018, a cross-sectional study was carried out in Zhongshan City, Guangdong Province, Southern China. All participants met the following inclusion criteria: (1) aged 65 years or above, (2) older Chinese people, (3) can communicate in Mandarin or Cantonese (e.g. can read Chinese or listen to Chinese); (4) living in community or long term care facility (i.e. service centre for older people in the community). Informed consent was received from the participants prior the interview and respective assessments.

### Study instruments

#### GFI

GFI was used to measure the frailty of older people which was developed by Steverink in the Netherlands [32]. It was a 15-item screening tool, all items of which were dichotomised to calculate GFI total scores. A higher GFI total score indicated a higher level of frailty [24, 35, 36]. GFI-C [20], as described before, was used as the studied instrument.

#### Simplified Barthel index (SBI)

The SBI (usually named Modified Barthel Index) was used to determine the degree of physical independence level of our participants. It has satisfactory psychometric properties among various groups of a population [38, 39]; Cronbach’s alpha values ranged from 0.953 to 0.965 [40, 41]. Interrater reliability was good with an intra-class correlation coefficient (ICC) value of 0.95–0.97 [38]. The predictive validity was demonstrated through correct prediction in the discharge outcomes among older people (i.e. community or residential care settings) using logistic regression analysis [42]. The optimal cut-off value of SBI for determining the categories of high dependency is below 12 (sensitivity 97.2%, specificity 97.4%) in older people with normal cognition [40]. This index in the Chinese version was used in hypothesis testing for the determination of construct validity in this study.

#### Abbreviated mental test (AMT)

The AMT was used to determine the cognitive level of our participants. It has the advantage of simplicity and brevity and has been widely used to screen impaired cognitive function in older people in Hong Kong [41, 43]. The best cut-off point is 7 (below 7 is considered cognitive impairment) with a sensitivity of 92.3% and specificity of 87.1% when used in older people in communities and nursing homes [43]. The reliability (Cronbach’s α = 0.814; ICC = 0.993) and validity (content validity, 0.92; concurrent validation, correlation with the Chinese Mini-Mental State Examination, *r* = 0.86; construct validity, known-groups method, t = 9.85, *p* < 0.001) were satisfactory according to a previous study [43]. The AMT Chinese version was used in hypothesis testing for the determination of construct validity in this study.

#### Fried’s frailty phenotype

Fried’s frailty phenotype was a clinical scale used for frailty diagnosis, which has been applied to multiple epidemiological studies and has predicted adverse clinical outcomes (i.e. mortality) [44–46]. This classification considers frailty by its physical characteristic or ‘phenotype’, which is assessed by the presence of at least three of the five parameters (weakness: low grip strength, slowness: slow walking speed, shrinking: unintentional weight loss of 4.5 kg or more in the previous year; exhaustion: low physical activity) [23]. Respondents without any of the parameters are non-frail, those meeting one or two parameters are classified as pre-frail, and those having three or more of the parameters are frail [47]. Sensitivity and specificity were well tested [29]. Since this scale served as the gold standard of frailty diagnostic tools to validate the other new frailty measurement in the literature [48–52], it was also used in the concurrent validation and diagnostic accuracy test in this study.

### Psychometric testing

The psychometric properties included reliability (i.e., internal consistency and stability), concurrent validity, and construct validity of the GFI-C were tested. For the establishment of construct validity, we applied the known-groups method, hypothesis testing, and confirmatory factor analysis (CFA) together (refer to Table 1 for the details).

### Diagnostic Acuracy test

Sensitivity and specificity analyses were used to indicate the diagnostic accuracy of the GFI-C, which included the precision and accuracy in screening frailty and pre-frailty community-dwelling older people [53]. The receiver-operating characteristic curve (ROC) was used to determine the optimal cut-off value of the GFI-C with reference to the frail and non-frail cases and the pre-frail and non-pre-frail cases determined by the gold standard (i.e. Fried’s frailty phenotype). A trained nurse conducted the entire frailty assessment to ensure consistency and creditability. Youden index measures the effectiveness of a diagnosis marker (i.e. Fried’s frailty phenotype) and enables the selection of an optimal threshold value (i.e. cut-off value) for it [54]. The area under the curve (AUC) was also computed to indicate the discriminative properties of the GFI-C cut-off value [55].

Sensitivity and specificity are equally important and should be greater than 0.70 for a valid screening tool used in the population-based study [55, 56].

### Statistical analysis

Most of the data were analysed using SPSS (version 24) except those of CFA, which were processed using AMOS (version 22). Descriptive statistics, including standard deviation (SD) and mean, were initially examined for continuous variables, and the frequency of distribution and percentage were reported for categorical variables. The variables were used for demographic description after data cleansing. As mentioned in the previous section on the psychometric testing plan, inferential statistics, including Cronbach’s α, ICC, Pearson product-moment coefficient of correlation, and independent sample t-test, were used appropriately to establish the reliability and validity of the GFI-C (Table 1). A *p*-value of 0.05 was accepted as significant.

## Results

### Characteristics of the participants

Of the 350 participants, nearly 70% (*n* = 240) were females. The ages ranged from 65 years to 93 years, with a mean of 75.27 (SD: 7.87). A majority of the study samples were from communities (*n* = 239, 68.3%), and the rest (*n* = 111, 31.7%) were from long term care facilities located in communities. Nearly 80% of the participants (*n* = 277) were married. Almost 30% (*n* = 96) were illiterate. Regarding financial status, 17.4% (*n* = 61) were economically independent. Over two-thirds (68.0%) had no religious belief, and 60% (*n* = 210) had a working experience.

Among the participants, about two-thirds (*n* = 237, 67.7%) had one or more co-morbidities. Hypertension (70.3%) and diabetes mellitus (22.8%) were the most common health problems among the older participants. On average, the numbers of daily drugs taken were 1.69 (SD 2.04). In general, over 80% of the study participants (*n* = 289) had not been hospitalised 1 year before the interview. Table 2 displays the demographic characteristics of the study participants.

**Table 2:** Demographic characteristics of the participants (*n* = 350)

Demographic characteristics	Overall
Age, mean (SD) Gender, n (%)	75.27 (7.87)
Male	110 (31.4)
Female	240 (68.6)
Recruitment source, n (%)	
Long term care facility	111 (31.7)
Community	239 (68.3)
Marital status, n (%)	
Married	277 (79.1)
Not married (single, divorced, widowed and others)	73 (20.9)
No. of children, mean (SD)	2.62 (1.62)
Education level, n (%)	
Illiterate	96 (27.4)
Primary school education	144 (41.1)
Secondary school education or above	110 (31.4)
Financial status, n (%)	
Economic independence	61 (17.4)
Dependence on relatives	74 (21.1)
Dependence on social endowment insurance	215 (61.4)
Religion, n (%)	
With religious belief	112 (32.0)
Without religious belief	238 (68.0)
Previous occupational status, n (%)	
No working experience	79 (22.6)
Housewife	61 (17.4)
Self-employed	32 (9.1)
Employed	178 (50.9)
No. of comorbidities, n (%)	
None	113 (32.3)
1	155 (44.3)
2	64 (18.3)
≥ 3	18 (5.1)
Prescribed with drugs, n (%)	
Yes	230 (65.7)
No	120 (34.3)
No. of daily drugs taken, mean (SD)	1.69 (2.04)
Hospitalised in past one year, n (%)	
Yes	61 (17.4)
No	289 (82.6)
GFI-C, n (%)^*^	
Non-frail	133 (38)
Prefrail	48 (13.7)
Frail	169 (48.3)
Fried’s frailty phenotype, n (%)	
Non-frail	59 (16.9)
Prefrail	147 (42)
Frail	144 (41.1)
Instrument, mean (SD)	
SBI (0-20)	18.29 (4.38)
AMT (0-10)	7.99 (3.04)

*AMT* Abbreviated Mental Test, *SBI* Simplified Barthel Index, *SD* Standard Deviation, ^*^The categories were based on the current results of diagnosis accuracy testing

### Psychometric testing

#### Reliability

The reliability results of the GFI-C were presented in terms of internal consistency and stability. The Cronbach’s α value of the GFI-C was 0.867 for the scale level and ranged from 0.687 to 0.755 for subscales, suggesting a satisfactory internal consistency. All the 50 invited participants completed the retest interviews (response rate = 100%). The value of the ICC was 0.865 (95% confidence interval (CI) = 0.774–0.921), which was regarded as satisfactory stability, and the ICC ranged from 0.441 to 0.792 among the subscales.

#### Validity

##### Concurrent validity

The concurrent validity of the GFI-C was examined by comparing the scores of the GFI-C and Fried’s frailty phenotype. The correlation between the total scores of the GFI-C and Fried’s frailty phenotype was 0.756 (*p* < 0.001), indicating significant correlation and optimal strength of correlation ( ≥ 0.7 and < 0.9) [55].

##### Construct validity

**Known-groups method** The total score of the GFI-C indicated that older people in long term care facility had significantly higher GFI-C scores (mean = 6.12; SD 4.05) than community-dwelling older people (mean = 2.44; SD 2.73; t = 8.26; *p* < 0.001).

**Hypothesis testing** The correlation between the total scores of the GFI-C and SBI was − 0.667 (*p* < 0.001), and that of the total scores of the GFI-C and AMT was − 0.774 (*p* < 0.001), indicating that both correlations were significant with sufficient strength and correct directional relationship [55]. Table 3 presents the detailed results of hypothesis testing.

**Table 3:** Correlation matrix of the GFI-C between the SBI and the AMT

	GFI-C			
	Total	Daily activities	Health problems	Psychosocial Functioning
SBI	−.667**	−.913**	−.346**	−.497**
AMT	−.774**	−.760**	−.529**	−.667**

*GFI-C* Groningen Frailty Indicator – Chinese, *SBI* Simplified Barthel Index, *AMT* Abbreviated Mental Test (Hong Kong version)

**p < 0.001

**Fig. 1:**
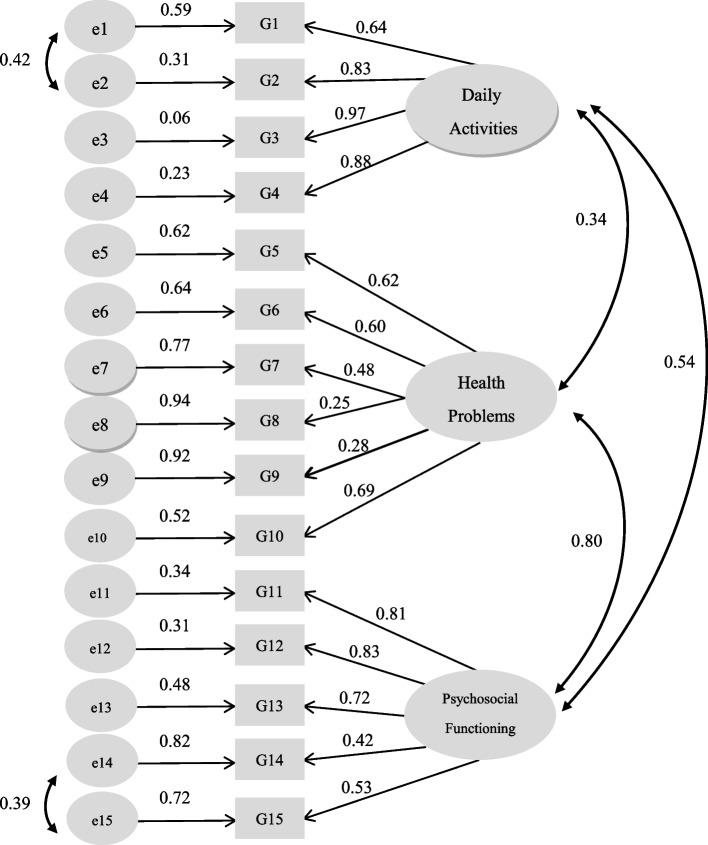
Confirmatory factor analysis model of the Groningen Frailty Indicator–Chinese version (GFI-C)

**CFA** Figure 1 lists the factor loading and parameter estimation of each item to the hypothesised subconstruct of the GFI-C. The results indicated that all the paths were significantly loaded to the hypothesised subconstructs (range of loadings = 0.25–0.97), and the factor loadings of 86.7% of items were greater than 0.32. The goodness-of-fit indices demonstrated an acceptable data model fitted with an χ^2^/df of 2.87, TLI of 0.92, CFI of 0.93, GFI of 0.92, RMR of 0.014, and RMSEA of 0.073. The findings suggested that the data of the GFI-C fitted well with a three-factor structure and provided additional evidence of its construct validity.

### Diagnostic accuracy test

**Fig. 2:**
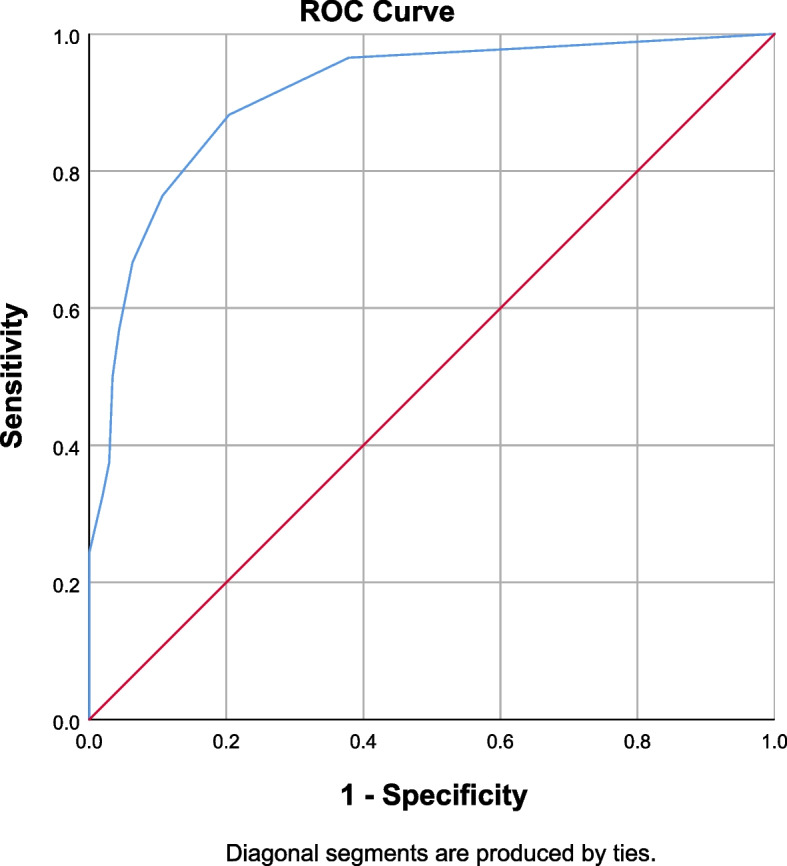
Area under the receiver operating characteristic (ROC) curve (AUC) for the GFI-C (*n* = 350) on frailty screening. Remark: GFI-C = Groningen frailty indicator–Chinese; ROC = Receiver-operating characteristic; AUC = Area under the curve; Frailty was diagnosed by a nurse using Fried’s Frailty Phenotype (FP). AUC=0.911 (95% CI = 0.880-0.942)

**Fig. 3:**
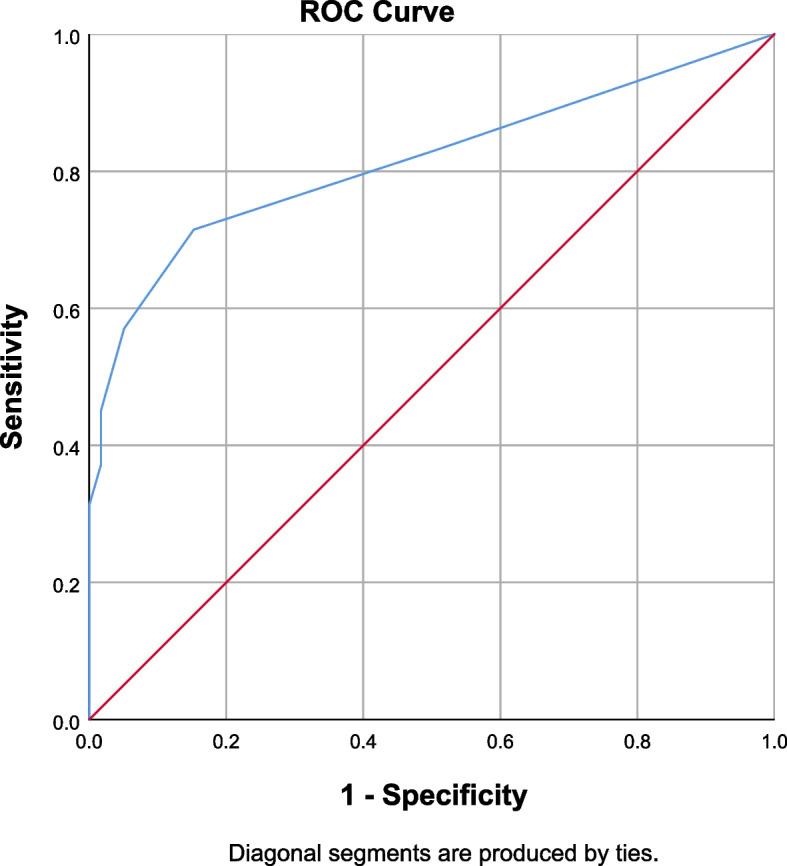
Area under the receiver operating characteristic (ROC) curve (AUC) for the GFI-C (n = 350) on pre-frailty screening. Remark: GFI-C = Groningen frailty indicator–Chinese*;* ROC = Receiver-operating characteristic; AUC = The area under the curve; Pre-frailty was diagnosed by a nurse using Fried’s Frailty Phenotype (FP). AUC=0.841 (95% CI = 0.767-0.861)

The sensitivity and specificity for the cut-off values of the GFI-C in relation to a gold standard (i.e. Fried’s frailty phenotype) were calculated and plotted in ROC curves (Figs. 2 and 3). Youden Index was calculated based on the sensitivity and specificity of the different cut-off values of the GFI-C scores. The largest value of Youden index was 0.678 for determining frailty, and the corresponding score of the GFI-C was ≤3, which indicated that the optimal cut-off value of the GFI-C was 3 (sensitivity = 88.2%, [95% CI: 81.8–93.0%]; specificity = 79.6%, [95% CI: 73.5–84.9%]). The values of sensitivity and specificity were both over 0.7, indicating that the cut-off is satisfactory for frailty screening. According to the ROC curve, the AUC was 0.911 (95% CI = 0.880–0.942), which indicated that the GFI-C had a good discriminative property in this study. For the screening of pre-frailty, the optimal cut-off value was 2 (sensitivity = 71.5%, [95% CI: 65.9–76.6%]; specificity = 84.7%, [95% CI: 73.0–92.8%]), which was still acceptable as reflected by the AUC (0.814) (refer to supplementary Table 1 and 2 for details).

## Discussion

The current results of the psychometric properties and diagnosis accuracy test of the GFI-C (refer to Table 4 for the details) enriched the applicability and utilization of this frailty scale in epidemiological research. The results demonstrated satisfactory psychometric properties of the GFI-C for assessing the frailty level of Chinese older people in long term care facility and the community. New cut-off values further enhanced the accuracy of screening of the frailty and pre-frailty status of Chinese older people, which added value to future population-based studies.

**Table 4:** Comparison of psychometric properties and diagnostic accuracy of the GFI-C with previously published results

	Results	Previous study results^a^
Reliability		
Internal consistency	Cronbach's α = 0.87	Cronbach's α = 0.68
Stability	ICC = 0.87, *p* < 0.001 (95% CI = 0.78–0.92)	*r* = 0.939 (*p* < 0.001)^b^
Validity		
Face validity	100% acceptable	84% of older persons had no difficulty completing the GFI
Content validity	CVI = 0.98	I-CVI = 0.83–1.0; S-CVI = 0.98 (S-CVI/UA = 0.66)^c^
Criterion-related validity	*r* = 0.76, *p* < 0.001	–
Construct validity		
1. Known-groups method	*t* = 8.71, *p* < 0.001 (95% CI = 2.95–4.52)	Statistically significant
2. Hypothesis testing		
Correlation with GFI-C and AMT score	*r* = −0.77, *p* < 0.001	The correlations for the convergent (0.45–0.61) and discriminant validity (0.08–0.50) were also as hypothesised.
Correlation with GFI-C and SBI score	*r* = − 0.67, *p* < 0.001	
3. Factor analysis	χ^2^/df = 2.87, TLI = 0.92, CFI = 0.93, GFI = 0.92, RMR = 0.014, RMSEA = 0.073	χ^2^ = 235.02, df = 84, (*p* < 0.00001), GFI = 0.98, RMR = 0.0063, RMSEA = 0.074^b^
Diagnostic accuracy		
Sensitivity for frailty	Cut-off value ≥3 Sensitivity = 88.2% (95% CI: 81.8–93.0%)	Cutoff value ≥4, Sensitivity = 66% (95% CI: 56–75%)^d^
Sensitivity for pre-frailty	Cut-off value ≥2 Sensitivity = 71.5% (95% CI: 65.9–76.6%)	Nil
Specificity for frailty	Cut-off value ≥3 Specificity = 79.6% (95% CI: 73.5–84.9%)	Cutoff value ≥4, Specificity = 87% (95% CI: 76–94%)^d^
Specificity for pre-frailty	Cut-off value ≥2 Sensitivity = 84.7% (95% CI: 73.0–92.8%)	Nil

*CI* Confidence Interval, *ICC* Intraclass Correlation Coefficient, *I-CVI*, Item-level Content Validity Index, *S-CVI* Scale-level Content Validity Index, *TLI* Tucker–Lewis Index, *CFI* Comparative Fit Index, *GFI* Goodness-of-fit Index, *RMR* Root Mean Square, *RMSEA* Root Mean Square Error of Approximation, *df* Degree of Freedom

^a^Previous study was based on Peters et al. (2012)

^b^This result was based on the previous study of Luh, Yu & Yang (2018)

^c^This result was based on the previous study of Xiang et al. (2019)

^d^This result was based on the previous study of Baitai et al. (2013)

### Issues in reliability

The Cronbach’s α of the total scores was 0.867, which indicated a good internal consistency [57]. This result was consistent with that of previous studies [34, 58, 59].

For the 2-week test-retest reliability, the result was good at the scale level (ICC = 0.865). However, it is noteworthy that this score is lower than that reported in a previous study. The subscale of ‘psychological functioning’ in the GFI might be affected by external factors.

In this study, a festival event, that is Chinese New Year, interweaved between the first and second interviews, which posed a plausible reason for inflating the retest score [60]. Chinese New Year means a new beginning and happiness to all Chinese people, and hence, participants in the retest interview may provide positive answers, particularly in the ‘psychosocial functioning’ subscale. Statistically, the percentages of agreement of items 14 and 15 were 74% and 76%, respectively, which were relatively low among all 15 items. Therefore, on one hand, it was anticipated that the current result might underestimate the stability of the GFI-C. On the other hand, the inflation of the retest score provided evidence that the GFI-C is sensitive to detecting the changes in the psychological and social condition under the frailty measurement. Further validation study should avoid the presence of festival events during the period of evaluation of test-retest reliability.

### Issues in validity

The results of the correlation matrix between the GFI-C and Fried’s frailty phenotype indicated that the two instruments had a significant and optimal correlation, showing satisfactory concurrent validity [55].

The literature accepted that Fried’s frailty phenotype can be served as a gold standard for validating the other frailty measurements [48, 49, 52, 61]. However, there were several cautions in the result interpretation [41, 62]. First, GFI-C is a self-reported type of questionnaire while Fried’s frailty phenotype is a clinical-based frailty assessment. Second, the former assessed four components of frailty including physical, psychological, cognitive and social conditions, which were recognised as important in frailty screening published in measurement review [14, 25]. Nevertheless, Fried’s frailty phenotype focused on the single dimension of physiological performance assessments. Lastly, the requirement of the assessor (laymen versus healthcare professionals) is different. These fundamental differences restricted the strength of magnitude of the correlation coefficient and an optimal value was recommended between 0.70–0.90 [41, 55]. Such results added credibility to support the concurrent validity of the GFI-C.

The theoretical hypothesis stated that the score of the GFI-C should be negatively correlated to the degree of cognitive level and level of physical independence of older people. The current results met and supported this hypothesis under the test of construct validity. With reference to Table 3, the strength of the correlation coefficient between GFI-C ‘Daily Activities’ and SBI presented the strongest. Besides, the correlation of the AMT with GFI-C ‘Daily Activities’ and ‘Psychological Functioning’ showed higher coefficients than that with ‘Health Problems’. These coefficients demonstrated that GFI-C subscales were convergent to the scales with high relevant concepts.

### Factor structure

The internal structure of a three-factor model of the GFI-C was further validated through CFA in this study, which was in line with Bielderman’s findings in the Netherlands [36].

By examining the factor loading of 15 items, all paths were significantly loaded onto the hypothesised subconstructs, and 86.7% of items obtained a loading of 0.32 or greater, except item 8 and 9 under the subconstruct of ‘Health Problems’. Indeed, with respect to the results of internal consistency, the corrected item–total correlation of items 8 and 9 was consistently low, indicating a weak homogeneity in the respective subconstruct.

Two pairs of error terms of items were co-varied: items 11 and 12, items 3 and 4, with large modification indices of 49.75 and 44.38, respectively. Given that large modification indices revealed the presence of factor cross-loadings and error covariance [63], model re-specification or modification was used, and the model was re-estimated for improvement of the model fit [57]. Such a method was commonly used in the literature for regulating the model fit [62–64].

In summary, the goodness-of-fit indices generated by the CFA model for the three-factor structure of the GFI-C were acceptable. All paths were significantly loaded to the hypothesised subconstructs. The evidence supported the construct validity of the GFI-C with three factors, namely ‘Daily Activities’, ‘Health Problems’ and ‘Psychosocial Functioning’.

### New cut-off values

By interpreting the results from ROC curves, a cut-off value of 2 (the maximum value of Youden Index) enriched the pre-frailty screening with the GFI-C, with acceptable sensitivity, specificity and AUC. This is new to the literature.

For frailty screening, a cut-off value of 3 provided satisfactory sensitivity (88.2%) and specificity (79.6%) compared with those of a previous study (cut-off value ≥4, sensitivity = 66%, specificity = 87%; Baitar et al., 2013). A conventional cut-off value of 4 for the GFI has been adopted in previous frailty epidemiological studies since the development of the instrument [24, 32, 34–36, 58, 59, 65]. A rare study re-examined the cut-off values of the GFI. However, the current study developed a new cut-off value for the Chinese population through the use of a gold standard of frailty measurement (i.e. Fried’s frailty phenotype), and the use of nurses in diagnostic procedures. The satisfactory and comparable sensitivity, specificity and the AUC results supported a new cut-off value of frailty screening. Three plausible reasons for such a change are discussed below.

First, in the background of Chinese Confucian ideology, the noun ‘face’ not only means the outside appearance of a person but also represents the self-esteem, dignity and reputation of a person and the invisible existence of social psychology in Chinese [41]. In item 5 of ‘what mark do you give yourself for physical fitness?’, Chinese people rated with a better fitness than Western people did because they may want to protect their ‘face’. Thus, for item 5, older Chinese people may obtain a lower GFI-C score (i.e. less frail) than older Western people with similar physical fitness.

Second, an old saying in Chinese mentions that ‘taking medications is just like taking poison’, which reflects the Chinese culture of not taking medications unless a person is really ill. Moreover, Chinese traditional herbal medicine is more acceptable in China than Western medicine. Although older people in China have to take medications, 91.8% of the community-dwelling older Chinese people were unaware of the names of medicines, and 55.6% had forgotten to take medications exactly as prescribed by their doctors [6]. Given that item 9 of the GFI-C inquired about the medication types of our participants, older people in China may fail to correctly distinguish the types of medications they are taking. In addition, they will not follow prescriptions occasionally and use Chinese herbal medicine or tea instead. Hence, their real medication status may be underestimated, which lowered the score of the GFI-C.

Last, a study published in 2010 stated that 22.8% of adults in China never measured their body weights, and the lower their education levels are, the higher the proportion of their weight gain is [66]. The demographic data of our participants showed that their average age was 75 years, and 27.4% of them were illiterate. Item 8 of the GFI-C was asking ‘during the past 6 months have you lost a lot of weight unwillingly?’ that required our participants to recognize their weight changes or have a habit of measuring their body weights regularly or recently. In this study, 92% of older people participants (*n* = 322) provided no weight loss answer to item 8, which may result in a low score of the GFI-C.

In the literature, frailty was strongly linked to the adverse outcomes of older people, including fracture, falls, hospital admission and mortality [51, 67–70]. The early detection of frailty can reduce adverse outcomes in older people, and thus, interventions for improving their health status can prevent them from becoming frail [14, 35, 36]. Moreover, frailty can be detected early with a GFI-C instrument. The results of sensitivity and specificity of the GFI-C (Table 4) showed that at a cut-off value of 3, sensitivity (i.e. 88.2%) was better than that in previous studies (sensitivity = 66%), but specificity (i.e. 79.6%) was lower (specificity = 87%) [36]. However, when the cut-off value was 4 (computed by the current database), the sensitivity decreased to 76.4% and the specificity increased to 89.3%. The high sensitivity decreased the number of false negatives of frailty, and a low specificity denoted a higher number of false positives of the GFI-C at a cut-off value of 3. Thus, the low sensitivity increased numerous false negatives of frailty, and a high specificity decreased the number of false positives at a cut-off GFI-C value of 4.

When applying the above concept in the actual situation of screening, the number of older people screened as frail was 169 at a cut-off value of 3 versus 132 at a cut-off value of 4. This result demonstrated that a cut-off value of 3 would be conservative to include more potential frail cases, which in turn reduced the chance of underdiagnosis. This attribute (i.e. higher sensitivity as priority) was important to a screening tool.

### Limitations

Apart from the satisfactory results of the current study, three areas of limitations deserved a discussion.

For generalisability, owing to convenience sampling and sample size, the results of the frailty prevalence rate cannot be generalised to the target population (i.e. all community-dwelling older people in China) [71]. However, this study aimed to validate the GFI-C but not investigate the prevalence rate.

It was also noted that the sex ratio of the whole Chinese population at 1.05:1.00 (Male vs Female) [4] while our samples demonstrated a significant sex imbalance (68.6% female versus 31.4% male). It may pose a risk of poor representativeness regarding the entire population. However, the average life expectancies of males and females are 73.64 and 79.43 years in China [4] and given the average age of 75.27 years old in our study samples, more females who still survived in older age should be expected. This phenomenon is widely reported in many gerontological studies [6, 40].

It was anticipated that some frail older people were unable to communicate. Due to the limitation of a self-reported scale, the assessment of frailty in this study required a process of interview and hence, non-communicable older people have been excluded. Therefore, the current results of diagnostic accuracy tests and new cut-off values may not be applicable to those non-communicable older people.

Fried’s frailty phenotype has been served as a gold standard for concurrent validation and diagnostic accuracy test in this study. It is noteworthy that the included domains of GFI are broader than that of Fried’s frailty phenotype, which may pose a risk of misdiagnosis or under-diagnosis. However, up to our best knowledge, there is no other option better than Fried’s in terms of creditability and recognition. Therefore, the result should be interpreted with that caution.

## Conclusion

The GFI-C is a validated and accurate tool for frailty status screening of community-dwelling older Chinese people. This study is the preliminary step for health providers to screen for frailty in China, and it can bring researchers closer to achieving a gold standard for diagnosing frailty. Using the self-reported GFI-C for screening the larger older population, which reaches over 200 billion in China nowadays, will help health providers in the rapid screening of frailty status among them. The early screening of frailty will receive adequate attention in gerontological nursing practice. Sustained efforts for interventional studies of the GFI-C and comparison of research results obtained from different parts of China or other Asia-Pacific regions may be useful in the development of the most suitable frailty instrument for older Chinese people.

## Supplementary Information

**Additional file 1: Supplementary Tables 1.** Sensitivity, specificity and Youden index for the GFI-C on frailty screening (*n* = 350). **Supplementary Tables 2.** Sensitivity, specificity and Youden Index for the GFI-C on pre-frailty screening (*n* = 350).

## Data Availability

The datasets generated and/or analysed during the current study are available from the corresponding author on reasonable request.
