# Supplementary material for: Groningen Frailty Indicator–Chinese (GFI-C) for pre-frailty and frailty assessment among older people living in communities: psychometric properties and diagnostic accuracy
Source: BMC Geriatr. 2022 Oct 7;22:788. doi: 10.1186/s12877-022-03437-1 (PMC9540721; doi:10.1186/s12877-022-03437-1)
Supplement: Supplementary file 1 — Additional file 1: Supplementary Tables 1. Sensitivity, specificity and Youden index for the GFI-C on frailty screening (n = 350). Supplementary Tables 2. Sensitivity, specificity and Youden Index for the GFI-C on pre-frailty screening (n = 350). [file 12877_2022_3437_MOESM1_ESM.docx]

Supplementary Tables 1 *Sensitivity, specificity and Youden index for the GFI-C on frailty screening (n = 350)*

| **GFI score** | **Sensitivity** | **Specificity** | **Youden Index** |
| --- | --- | --- | --- |
| ≤1 | 0.979 | 0.364 | 0.343 |
| ≤2 | 0.965 | 0.621 | 0.587 |
| ≤3 | 0.882 | 0.796 | 0.678 |
| ≤4 | 0.764 | 0.893 | 0.657 |
| ≤5 | 0.667 | 0.937 | 0.604 |
| ≤6 | 0.569 | 0.956 | 0.526 |
| ≤7 | 0.500 | 0.966 | 0.466 |
| ≤8 | 0.375 | 0.971 | 0.346 |
| ≤9 | 0.326 | 0.981 | 0.307 |
| ≤10 | 0.243 | 1.000 | 0.243 |
| ≤11 | 0.160 | 1.000 | 0.160 |
| ≤12 | 0.090 | 1.000 | 0.090 |
| ≤13 | 0.042 | 1.000 | 0.042 |
| ≤14 | 0.014 | 1.000 | 0.014 |
| ≤15 | 0.007 | 1.000 | 0.007 |
| ≤16 | 0.000 | 1.000 | 0.000 |

*Remark:*

GFI-C = Groningen Frailty Indicator–Chinese version;

Frailty was diagnosed by a nurse using Fried’s Frailty Phenotype (FP).

Supplementary Tables 2 *Sensitivity, specificity and Youden Index for the GFI-C on pre-frailty screening (n = 350)*

| **GFI score** | **Sensitivity** | **Specificity** | **Youden Index** |
| --- | --- | --- | --- |
| ≤1 | 0.832 | 0.492 | 0.323 |
| ≤2 | 0.715 | 0.847 | 0.562 |
| ≤3 | 0.570 | 0.949 | 0.520 |
| ≤4 | 0.450 | 0.983 | 0.433 |
| ≤5 | 0.371 | 0.983 | 0.354 |
| ≤6 | 0.313 | 1.000 | 0.313 |
| ≤7 | 0.271 | 1.000 | 0.271 |
| ≤8 | 0.206 | 1.000 | 0.206 |
| ≤9 | 0.175 | 1.000 | 0.175 |
| ≤10 | 0.120 | 1.000 | 0.120 |
| ≤11 | 0.079 | 1.000 | 0.079 |
| ≤12 | 0.045 | 1.000 | 0.045 |
| ≤13 | 0.021 | 1.000 | 0.021 |
| ≤14 | 0.007 | 1.000 | 0.007 |
| ≤15 | 0.003 | 1.000 | 0.003 |
| ≤16 | 0.000 | 1.000 | 0.000 |

*Remark:*

GFI-C = Groningen Frailty Indicator–Chinese version;

Frailty was diagnosed by a nurse using Fried’s Frailty Phenotype (FP).
